# Behavioral and Biochemical Assays for Autism Models of Wistar Rats

**DOI:** 10.7759/cureus.52066

**Published:** 2024-01-10

**Authors:** Kavitha Ukkirapandian, Kayalvizhi Elumalai, Karthika Priyadharshini Udaykumar, Sundaravadivel VP, Muthulakshmi Rangasmy

**Affiliations:** 1 Physiology, Meenakshi Medical College Hospital and Research Institute, Meenakshi Academy of Higher Education and Research (MAHER) University, Kanchipuram, IND; 2 Physiology, Jawaharlal Institute of Postgraduate Medical Education and Research, Pondicherry, IND

**Keywords:** sodium valproate, biochemical changes, behavior, autism, animal model

## Abstract

Being a "behavioral disorder," autism spectrum disorder (ASD) is difficult to manage because its precise etiology is uncertain. In order to better understand the pathophysiology of autism and explore various therapeutic approaches, animal models are developed. Animal models of autism caused by valproate during pregnancy exhibit strong construct validity and reliability. Hence, this study was done among autism-induced rats with the aim of identifying the behavioral and biochemical assays. Pregnant rats were administered sodium valproate on the 12th day of gestation, while control pregnant rats received normal saline. The rats' offspring that received normal saline during intrauterine life were grouped as control, and the rats' offspring that received valproate were grouped as autism-induced. From postnatal day (PND) 21, behavioral assessments were done by using the Y maze (repetitive behavior) and the T maze (social behavior). The estimation of antioxidant profile (malondialdehyde {MDA}, glutathione {GSH}, catalase (CAT), and superoxide dismutase {SOD}), proinflammatory markers (tumor necrosis factor {TNF} alpha, transforming growth factor {TGF} beta, interleukin {IL} 6, and IL-1 beta), neurotransmitters (gamma-aminobutyric acid {GABA} and serotonin), and brain-derived neurotrophic factor (BDNF) in the hippocampal region was done. Oxidative stress, increased proinflammatory markers, and increased serotonin were recorded in the autism group. Rats with autism had a significant decrease in GABA and BDNF levels. These biochemical alterations can be correlated with clinical features of autism to diagnose and manage the disorder at the earliest.

## Introduction

The neurodevelopmental illness known as autism spectrum disorder (ASD) results in a wide range of aberrant behaviors in its patients, including poor speech, social impairment, and repetitive behavior. According to the WHO (2021), one in 160 children worldwide have ASD, and one in 44 children in the United States have the disorder [1]. Even though developed countries are the only ones where the prevalence of ASD is heavily reported, the actual situation may differ. The disorder's intensity is like an iceberg because of ignorance. Apart from that, autism encompasses a broad variety of limitations and requirements that signify different levels of disability and treatment requests.

Since the precise pathophysiology underlying ASD is unknown, there is no biomarker to confirm the diagnosis. Therefore, animal models of autism were developed in order to study in detail the neuropathological changes and to identify the important biochemical markers related to ASD. Various prenatal and postnatal models of autism were studied and grouped under genetic and epigenetic models. Previous studies reported that autism behavioral patterns in rat offspring were produced by valproate exposure during the prenatal period (E10-E12), which occurs before neural tube closure and roughly corresponds to the "first trimester" of human embryonic life [2].

Valproate, which causes oxidative stress and altered redox processes that have been shown in both children [3] and rats [4], is more harmful to the embryonic brain. Excitatory/inhibitory neurotransmitter imbalance is a commonly recognized theory in ASD based on clinical and preclinical findings. Neurotrophic variables need to be tightly controlled for brain homeostasis [5]. Therefore, altered biochemical changes require careful investigation. This study was undertaken to identify the significant biochemical changes in autism-induced rats for the early diagnosis of ASD, which helps in better diagnosis.

## Materials and methods

The animal model of autism was created after getting the Institutional Animal Ethical Committee clearance of the Meenakshi Medical College Hospital and Research Institute (MMCHRI) (protocol number: 001/2019-20). This work was carried out in the Department of Physiology at the Meenakshi Medical College Hospital and Research Institute. In female rats, after mating, conception was confirmed in the morning the next day via vaginal examination. At gestational day 12, the pregnant rats were given an injection of sodium valproate in the dose of 500 mg/kg intraperitoneally to induce autism, and the control group of pregnant rats was given normal saline: group 1, rat pups of normal saline-exposed rats (n=10); group 2, rat pups of valproate-induced autism rats (n=10).

All the rat pups were allowed to stay with their mother rats for 15 days after birth without any physical interruption in order to maintain a maternal bond. Behavioral analysis was performed by using the T maze and Y maze to assess the social and repetitive behavior, respectively.

Y maze test

The purpose of this test was to evaluate repetitious behavior. The three arms of the Y maze are A, B, and C. Animals were positioned in the middle of the device for five minutes, and admissions into each arm were recorded. A, B, and C visits in succession were counted as a single trio in order to assess spontaneous alternation behavior. When an animal enters the first arm after the second arm (e.g., A, B, and A) without changing, it is interpreted as a false visit. Perseverance was interpreted as the first and second entry into the same arm (e.g., A and A) [6]: $Probability \, of \, spontaneous\, slterations= \frac{Number\, of\,triads}{Total\, arm\, entries\, -2}$, $Probability \, of \, incorrect\, visits=\frac{Number\, of\, incorrect \, visits}{Total\, arm\, entries}$, and $Probability \, of \,perseverations=\frac{Number\, of\, perseverations}{Total\, arm\, entries}$.

T maze

The T maze, which contains three chambers, was used to assess social behavior. The animals under test were placed in the center chamber, with the remaining two arms being utilized for testing. The animal was given five minutes to become acclimated to the equipment before being tested for social preference behavior for an additional five minutes. This was accomplished by placing an unfamiliar animal in one arm while leaving the other vacant. The test animal was then left in the center arm for five minutes. All three arms' total time was tallied [7].

The social preference test was followed by the social novelty test. Here, a test animal was left in the center arm, an elderly stranger was placed in the same arm, and a new stranger was placed in an empty chamber. All three arms' total time was tallied.

Rat pups were scarified at the end of five weeks or approximately the 36th day of postnatal day (PND) (before sexual maturity, which is six weeks), and biochemical markers in the hippocampus region of the brain were measured.

Biochemical analysis

Malondialdehyde (MDA)

A thiobarbituric acid reactive substance (TBARS) assay kit was used. Reaction mixtures were made by adding 1 mL of tissue homogenate (sample), sodium dodecyl sulfate (SDS), 1 mL of 2.8% acetic acid solution, and 1.5 mL of 0.37% of thiobarbituric acid (TBA) in 50 mM NaOH. This reaction mixture was boiled for 20 minutes in a water bath to develop an MDA-TBA colored compound. Centrifugation was done at the speed of 1500 rpm for two minutes. After centrifugation, the supernatant was aspirated, and a pink-colored compound was read against a blank in a spectrophotometer at 532 nm. Values were compared with a standard curve. MDA was expressed as nmol/mg of protein.

Superoxide Dismutase (SOD)

To assess the enzymatic antioxidant SOD, the reaction mixture was made by adding 1.2 mL sodium pyrophosphate (SPP), 0.1 mL phenazine methosulphate (PMS), 0.3 mL nitro blue tetrazolium (NBT), and 2.8 mL H_2_O; 0.2 mL of nicotinamide adenine dinucleotide hydrogen (NADH) was added to initiate the reaction. This mixture was incubated at 30°C for two minutes, and 1 mL of glacial acetic acid was added to arrest the reaction. Four milliliters of n-butanol was added to the mixture, and after vigorous shaking, it was allowed to stand for 10 minutes. The intensity of the colored compound was read at 560 nm in a spectrophotometer. SOD was expressed as µmol/minute/mg of protein.

Glutathione (GSH)

To assess the nonenzymatic antioxidant GSH, the reaction mixture was made by adding 1.5 mL sample (tissue homogenate) and 2 mL trichloroacetic acid (TCA, 5%). After centrifugation, the supernatant was mixed with 1 mL of Ellman's reagent (5,5′-dithiobis(2-nitrobenzoic acid)) and 4 mL disodium hydrogen phosphate. The yellow-colored compound developed was read at 412 nm by using a spectrophotometer. Glutathione was expressed as nmol/mg of protein.

Proinflammatory Marker

Tumor necrosis factor (TNF) alpha was assessed by using the sandwich enzyme-linked immunosorbent assay (ELISA) method. Procedures were followed based on the manufacturer's instructions.

A standard solution was taken in two wells side by side 100 μL each, and the wells were covered and incubated for 90 minutes at 37°C. After removing the liquid, immediately, a "biotinylated detection antibody" solution of 100 μL was added to each well and covered with the plate sealer and incubated for one hour at 37°C. The solution was decanted, and 350 μL of "wash buffer" was added to each well. After 1-2 minutes, again, the solution was decanted from the wells, and the washing step was repeated three times. "Horseradish peroxidase (HRP) conjugate working solution" was added to each well. After covering, it was incubated for 30 minutes at 37°C. The solution was decanted, and the washing steps were repeated five times as conducted. "Substrate reagent" was added to each well. After covering, it was incubated for 15 minutes at 37°C. A stop solution of 50 μL was added to each well. The optical density (OD) value was determined for each well at once at 450 nm by using a microplate reader.

Serotonin was assessed by the competitive ELISA method. Procedures were followed based on the manufacturer's instructions.

Serotonin was quantitatively acylated. After that, 25 µL of the acylated solution "standards, controls, and samples" were added into the appropriate wells of the serotonin microtiter strips; 100 µL of the "serotonin antiserum" was added into all wells. The microtiter plate was covered and incubated at room temperature for 30 minutes. Serotonin microtiter was shaken well and incubated for one hour at room temperature. Microtiter wells were washed by using 300 µL wash buffer, and 100 µL conjugate was added to the wells and incubated for 15 minutes at room temperature, on a shaker for 600 rpm. After shaking it again, it was incubated for 15 minutes at room temperature. The washing steps were repeated, and 100 µL of substrate was added. Incubation was repeated on a shaker at room temperature for 15 minutes, and a stop solution of 0.25 M H_2_S0_4_ was added to each well and was shaken properly. The colored compound formed was read at 450 nm.

Gamma-aminobutyric acid (GABA) was assessed by competitive ELISA assay. Procedures were followed based on the manufacturer's instructions.

Detector A reagent was added to the well for a blank, standard, and sample. After adding reagent A, they were shaken well by using a microplate shaker and incubated for one hour at 37°C. The solution was aspirated, and the wells were washed by using a wash buffer. After three times of washing, these wells were dried. Detector reagent B of 100 µL was added and incubated for one hour. The washing steps were repeated for 15-20 minutes. A substrate solution was added and incubated for 15 minutes at room temperature, which produced a yellow-colored compound. After the washing steps, a stop solution was added, and a colored compound concentration was read at 450 nm by spectrophotometer.

Brain-derived neurotrophic factor (BDNF) was estimated by using respective ELISA kits. Procedures were followed based on the manufacturer's instructions.

One hundred microliters of standard and sample solution were added to the wells. After covering the microtiter plate, it was incubated for 90 minutes at room temperature. The wells were washed by using a wash buffer. After drying the wells, 100 µL of biotin-labelled detection antibody was added and incubated for 60 minutes at 37°C. The washing steps were done by using a wash buffer, and the wells were dried; 100 µL of streptavidin-HRP working solution was added into each well and incubated at 37°C for 45 minutes. The washing steps were done by using a wash buffer, and the wells were dried. One hundred microliters of 3,3′,5,5′-tetramethylbenzidine (TMB) substrate solution was added into each well and incubated for 30 minutes at 37°C; 100 µL of stop solution was added, and a yellow-colored compound was formed. It was read at 450 nm by using a spectrophotometer.

Statistical analysis

The data was presented as mean±standard error. Student's unpaired t-test and one-way analysis of variance (ANOVA) were done by using GraphPad Prism 8 (GraphPad Software, San Diego, CA) to assess the statistical significance, and a P value was fixed at 0.05 as significant.

## Results

Y maze

Student's t-test showed a decrease in the percentage of spontaneous alternation and a significant increase in the percentage of incorrect visit and perseveration in the autism group when compared to the control group, which indicates the repetitive behavior in autism (Table 1).

**Table 1:** Y maze: assessment of repetitive behavior SE: standard error

Y maze	Control (mean±SE)	Autism (mean±SE)	P value
Probability of spontaneous alternation	35.26±3.54	22.84±3.61	0.0093
Probability of incorrect visit	9.75±1.30	17.84±1.89	0.0055
Probability of perseveration	0.50±0.34	9.17±2.14	0.0025

T maze

In the T maze, control group rats spent more time with a stranger rat than empty chambers, which indicates social behavior of rats, whereas autism-induced rats spent more time in empty chambers than a stranger rat, which indicates social isolation (Table 2).

**Table 2:** T maze: social preference behavior SE: standard error

T maze (seconds)	Control (mean±SE)	Autism (mean±SE)
Time spent in the empty chamber	85.00±5.00	116.67±8.43
Time spent in the center chamber	60±19.15	98.33±7.03
Time spent in a stranger 1	155±18.03	85±8.47
P value	0.0016	0.0420

In the T maze social novelty behavior test, control rats spent more time with stranger 2 rat, followed by stranger 1 rat and the center chamber, which showed social novelty behavior of rats. The autism-induced group spent more time in the center chamber than the other two chambers, which indicates altered social behavior (Table 3).

**Table 3:** T maze: social novelty behavior SE: standard error

T maze (seconds)	Control (mean±SE)	Autism (mean±SE)
Time spent in the center chamber	46.67±5.34	123.33±2.28
Time spent in stranger 1	105±4.05	105±2.53
Time spent in stranger 2	140±6.99	71.67±4.52
P value	0.0008	0.0013

MDA level was significantly increased, and GSH, catalase (CAT), and SOD levels were significantly reduced in the autism-induced group when compared to the control group. The proinflammatory markers TNF alpha, transforming growth factor (TGF) beta-1, interleukin (IL) 6, and IL-1 beta were significantly elevated in the autism-induced group compared to the control group. Serotonin and GABA levels were significantly increased in the autism-induced group than the control group. The BDNF was significantly reduced in the autism-induced rats (Table 4).

**Table 4:** Biochemical parameters MDA, malondialdehyde; GSH, glutathione; SOD, superoxide dismutase; CAT, catalase; TNF, tumor necrosis factor; TGF, transforming growth factor; IL, interleukin; GABA, gamma-aminobutyric acid; BDNF, brain-derived neurotrophic factor; SE, standard error

Biochemical parameters	Control (mean±SE)	Autism (mean±SE)	P value
MDA (nm/mg of protein)	7.67±0.51	12.13±1.06	0.0035
GSH (nmol/mg of protein)	21.91±0.99	13.55±2.71	0.0057
CAT (µmol/minute/mg of protein)	8.55±0.42	3.26±0.3171	<0.0001
SOD (µmol/minute/mg of protein)	6.71±0.32	3.13±0.2971	<0.0001
TNF alpha (pg/mg of protein)	299.5±3.3	407±26.53	0.0024
TGF beta (pg/mg of protein)	278.3±6.0	385.0±9.2	0.0001
IL-6 (pg/mg of protein)	46.3±4.9	62±8.7	0.0003
IL-1 beta (pg/mg of protein)	56.5±4.0	67.8±7.7	0.0003
Serotonin (ng/mL)	122.67±5.08	137.33±2.94	0.0315
GABA (ng/mL)	389.16±11.72	239.16±24.30	0.0002
BDNF (pg/mg of tissue)	5.98±0.21	4.31±0.29	0.0011

## Discussion

Rat pups exposed to valproate were set as a prenatal valproate-induced animal model, and the T and Y mazes were used to identify autistic features in the animals. Repetitive behavior was observed among autism-induced rats in the Y maze; this was indicated by a decrease in the proportion of spontaneous alternations and a rise in the percentage of perseverations and erroneous visits. These results were supported by Hu (2019) [8]. A three-chamber T maze test was employed in this investigation to evaluate social novelty and preference behaviors. Rats with autism did not interact socially in the social preference or social novelty tests. The biochemical analysis validated these modified behavioral characteristics.

Elevated oxidative stress has been connected to the genesis of certain neurodegenerative diseases, such as Parkinson's and Alzheimer's. Oxidative stress has also been linked to ASD in a number of studies [9,10]. The results of this study are also in line with earlier research, which found that the autism-induced group's hippocampal GSH, CAT, and SOD levels were decreased and their MDA levels were raised, indicating lipid peroxidation [11]. Bowers et al. (2011) discovered a genetic variance in children with ASD's glutathione-like antioxidant system. In this study, valproate may have changed the genes governing the antioxidant system through its epigenetic properties, leading to increased oxidative stress in the group with autism [12].

In this study, the autism-induced group showed a considerable rise in the proinflammatory marker TNF alpha in the hippocampal region as compared to the control group. These outcomes agreed with earlier research by Deckman et al. (2018) [13]. The study's findings of elevated cytokines and proinflammatory markers in the autism-induced group indicate that autism is associated with altered neuroinflammation. Young et al. (2011) revealed that children with ASD have significantly higher levels of nuclear factor kappa B (NFkB) activation than usual children, which is concurrent with our findings [14].

5-Hydroxytryptamine (5-HT) has a major role in the maturation of brain cells. Serotonergic neurons innervate a broad range of brain areas, which enables 5-HT to influence the neural circuitry governing a spectrum of behavior. This study's findings showing valproate-induced autism had considerably higher levels of brain serotonin were consistent with a recent study [15]. Previous research on children with autism has also shown a correlation between autism and elevated serum 5-HT levels [16,17]. So, hyperserotonemia may serve as a potent indicator of autism.

In late embryonic/early postnatal stages, GABA, the main inhibitory neurotransmitter in adults, has been shown to have an excitatory function. This function depolarizes and stimulates the targeted cell by an outwardly directed flow of chloride [18]. Numerous developmental processes, such as cell migration, differentiation, and synapse formation, are regulated by GABA's depolarizing impact and the subsequent calcium influx [19]. In agreement with our findings, brain samples from individuals with ASDs have also revealed changes in gamma-aminobutyric acid A (GABAA) and gamma-aminobutyric acid B (GABAB) receptors and the mRNA encoding for the glutamic acid decarboxylase (GAD) 65 and GAD67 enzymes [20-22].

Tyrosine kinase B (TrkB) receptor is bound by BDNF, which then uses phosphoinositide phospholipase C-gamma (PLC-γ), GTPases of the Rho family pathway, and phosphatidylinositol 3-kinase (PI3K) to mediate neuronal survival. Since BDNF is necessary for the development and operation of neurons, reduced levels in the autism group as reported in this study may lead to aberrant synapse formation, which manifests as a neurodevelopmental disorder [23]. These findings demonstrated that prenatal valproate exposure resulted in behavioral abnormalities, as well as other autistic traits in the offspring of rats. These effects were caused by elevated oxidative stress, aberrant immune system stimulation during development, imbalance in neurotransmitter level, and reduced neurotrophic factor, BDNF.

The limitation of this study is only selective biochemical parameters, which were studied if related to autism. In the future, we plan to examine other relevant biochemical parameters and their mRNA expression in autism.

## Conclusions

In this study, the alteration of biochemical parameters among autism-induced small animals was well correlated with autism clinical features. Children with autism should also be investigated carefully, and their clinical features must be associated with the biochemical parameters including antioxidant level, proinflammatory markers, GABA, serotonin, and BDNF to manage the disorder systematically.
